# Disruption of the Metal Ion Environment by EDTA for Silk Formation Affects the Mechanical Properties of Silkworm Silk

**DOI:** 10.3390/ijms20123026

**Published:** 2019-06-21

**Authors:** Qingsong Liu, Xin Wang, Xiaoyin Tan, Xiaoqian Xie, Haonan Dong, Xinning Li, Yi Li, Ping Zhao, Qingyou Xia

**Affiliations:** 1 Biological Science Research Center, Southwest University, Chongqing 400716, China; liuqingsong@email.swu.edu.cn (Q.L.); swuwangxin@swu.edu.cn (X.W.); xiaoyintan@email.swu.edu.cn (X.T.); Xiaoqian123@email.swu.edu.cn (X.X.); HaonanDong.1995@gmail.com (H.D.); lixinning124@live.com (X.L.); yili89716@gmail.com (Y.L.); zhaop@swu.edu.cn (P.Z.); 2 Chongqing Key Laboratory of Sericultural Science, Chongqing 400716, China; 3 Chongqing Engineering and Technology Research Center for Novel Silk Materials, Chongqing 400716, China

**Keywords:** metal ion, silk fiber, mechanical properties

## Abstract

Silk fiber has become a research focus because of its comprehensive mechanical properties. Metal ions can influence the conformational transition of silk fibroin. Current research is mainly focused on the role of a single ion, rather than the whole metal ion environment. Here, we report the effects of the overall metal ion environment on the secondary structure and mechanical properties of silk fibers after direct injection and feeding of silkworms with EDTA. The metal composition of the hemolymph, silk gland, and silk fiber changed significantly post EDTA treatment. Synchrotron FTIR analysis indicated that the secondary structure of silk fiber after EDTA treatment changed dramatically; particularly, the β-sheets decreased and the β-turns increased. Post EDTA treatment, the silk fiber had significantly decreased strength, Young’s modulus, and toughness as compared with the control groups, while the strain exhibited no obvious change. These changes can be attributed to the change in the metal ion environment in the silk fibroin and sericin in the silk gland. Our investigation provides a new theoretical basis for the natural silk spinning process, and our findings could help develop a method to modify the mechanical properties of silk fiber using metal ions.

## 1. Introduction

Silk fiber is a natural polymer material [1]. Recently, silk fiber has received extensive attention because of its outstanding mechanical properties such as high fracture strength, high toughness, and exceptional extensibility [2]. Modifying the mechanical properties of silk fiber in order to expand their application in various fields has attracted the interest of many researchers. Various methods have been used to improve the mechanical properties of silk fiber. A number of researchers have reported that the mechanical properties of silk fiber can be modified by transgenic silkworm overexpression with the spider gene [3,4,5,6]. In addition, numerous studies have attempted to endow the silk fiber with special mechanical performance directly by feeding silkworms with inorganic nanoparticles such as single-walled carbon nanotubes, nanocarbon, TiO_2_ nanoparticles, and graphene [7,8,9,10]. Especially, recent evidence suggests that metal ions, pH, and shear forces are involving in the conformational transition of silk proteins, which will affect the mechanical properties of silk fiber [11,12]. Studies have shown that Mg^2+^, Cu^2+^, Fe^3+^, and Zn^2+^ can induce the conformational transition of silk fibroin from random coil/helix to β-sheet in vitro [13,14,15,16,17]. Ca^2+^ can induce the formation of a stable protein network in silk fibroin, while Na^+^ and K^+^ are thought to break down the protein network in silk fibroin [15,17]. However, injection of certain metal salt solutions such as CaCl_2_, KCl, CuCl_2_, and FeCl_3_ can improve the mechanical properties of silk fiber [16,17,18]. Furthermore, the mechanical properties of silk fiber can also be modified by genetically disrupting the Ca^2+^ content during silk fiber formation [19].

Although previous studies have suggested that metal ions can impact the structure of the regenerative silk fibroin (RSF), they have usually focused on one or two types of metal ions without changing the content of multiple metal ions simultaneously. Ethylenediaminetetraacetic acid (EDTA) is a good chelating agent [20,21], which can form a highly soluble complex with a wide variety of metal ions in an aqueous solution [22,23]. Therefore, we aimed at altering the contents of multiple ions through EDTA to disturb the overall metal ion environment simultaneously, to further explore the effects of the overall metal ion environment on the mechanical properties of silk fiber.

In this study, we directly injected EDTA (IEDTA) and fed EDTA (FEDTA) to silkworms to disturb the overall metal ion environment in the silk fiber formation, and observed its effects on the secondary structure and mechanical properties of the silk fiber. This study will deepen our understanding of the relationship between the overall metal ion environment and the mechanical properties of silk fiber. It will also provide information on how the mechanical properties of silk fiber can be improved by altering the metal ion environment.

## 2. Results and Discussion

### 2.1. Observation of Morphological Characteristics

**Figure 1:**
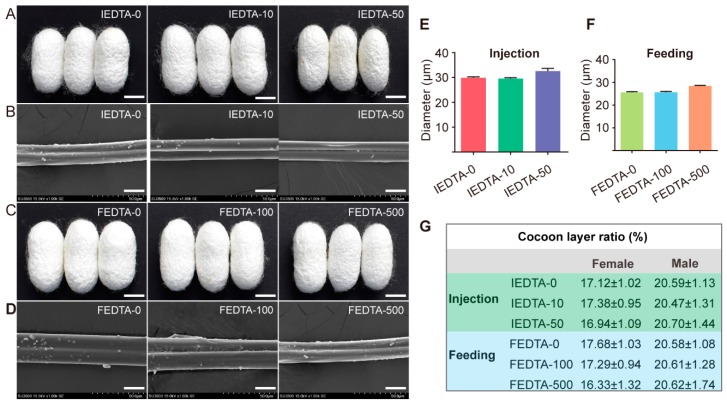
Visual appearance of cocoons and silk fiber after EDTA injection and feeding. (**A**,**C**), cocoon shapes after EDTA injection and feeding, scale bar is 1 cm. (**B**,**D**), SEM image of IEDTA and FEDTA silk fiber, scale bar is 20 μm. (**E**,**F**), average diameter of silk fiber after EDTA treatment. (**G**) Cocoon layer ration of silkworms after EDTA injection and feeding. IEDTA-0: Injecting 0 mM EDTA to silkworms, IEDTA-10: Injecting 10 mM EDTA to silkworms, IEDTA-50: Injecting 50 mM EDTA to silkworms; FEDTA-0: Feeding 0 mM EDTA to silkworms, FEDTA-100: Feeding 100 mM EDTA to silkworms, FEDTA-500: Feeding 500 mM EDTA to silkworms.

To investigate the effects of the overall metal ion environment on the mechanical properties of silk fiber, we injected and fed EDTA to the fifth instar larvae of silkworm until spinning. All the silkworms survived the treatment process. We first observed the morphology of cocoons after injection and feeding. As shown in Figure 1A,C, we found that EDTA showed no effect on the shape and size of cocoons, compared with those of control groups. Moreover, scanning electron microscope (SEM) imaging was used to study the morphology of silk fiber. While comparing the EDTA treated silk fiber with the control silk fiber, we observed that EDTA had no effect on the morphology of the silk fiber (Figure 1B,D). Furthermore, we measured the diameter of silk fiber by SEM. The statistical results showed that the diameter of the silk fiber did not change significantly after injection and feeding (Figure 1E,F). All these results demonstrated that the EDTA had little effect on the morphological characteristics of cocoons and single silk fiber. On the other hand, when comparing the cocoon layer ratio of EDTA treated silkworms with the control group (Figure 1G), the EDTA does not significantly affect this ratio, which is important in economic valorization of the cocoons.

### 2.2. EDTA Disturbed the Ionic Environment for Silk Fiber Formation

**Figure 2:**
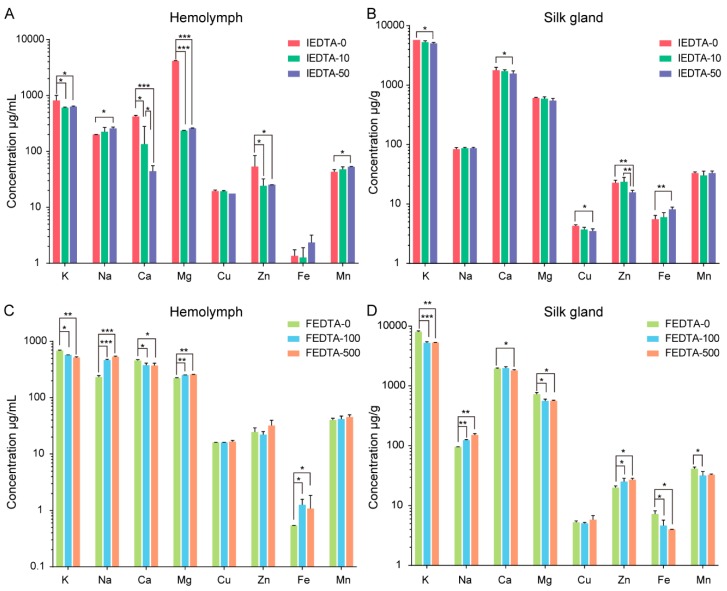
The contents of metal elements in the hemolymph and the silk gland after treatment with EDTA. (**A**) Hemolymph after being injected with EDTA. (**B**) Silk gland after being injected with EDTA. (**C**) Hemolymph after being fed with EDTA. (**D**) Silk gland after being fed with EDTA. Error bars, SD; * *p* < 0.05, ** *p* < 0.01, *** *p* < 0.001 (One-way ANOVA). *y*-Axis data was processed by log 10.

In order to confirm the effects of EDTA in vivo, we first measured the content of metal elements in the hemolymph and silk gland in silkworms (wandering stage). As shown in Figure 2, we tested eight kinds of metal elements, including Na, K, Ca, Mg, Fe, Zn, Cu, and Mn. Figure 2A,B shows that the contents of the metal elements in the hemolymph and the silk gland changed significantly after injection of EDTA, especially for K, Na, Ca, Mg, and Zn, whose ion contents changed dramatically in the IEDTA hemolymph. Additionally, a significant change was observed in the hemolymph and silk gland post EDTA treatment (Figure 2C,D). The Na, K, Ca, Mg, Fe, Zn, and Mn ion contents in the FEDTA silk gland increased or decreased significantly, which indicated that our treatment was effective. The present results have demonstrated that both injection and feeding can disturb the overall metal ion environment in the hemolymph and the silk gland.

**Figure 3:**
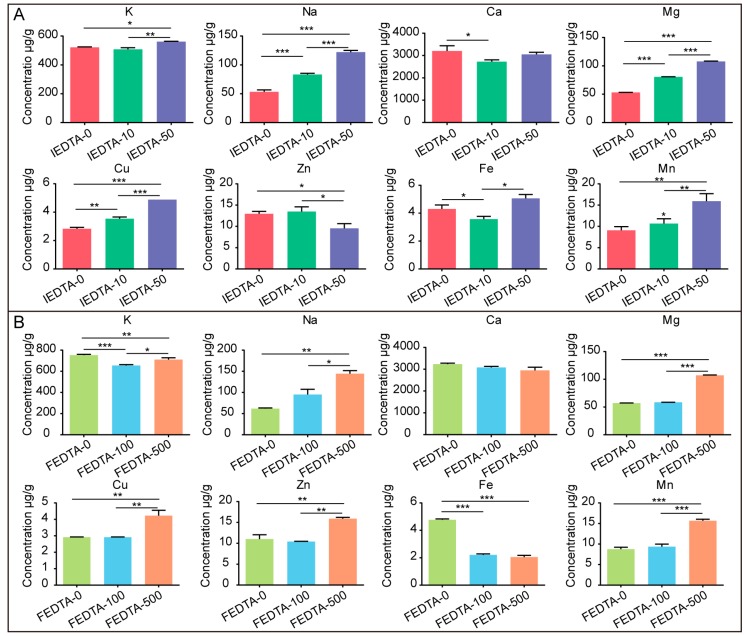
The content of metal elements in silk fiber after treatment. (**A**) The EDTA silk fiber after injection; (**B**) The EDTA silk fiber after feeding. Error bars, SD, * *p* < 0.05, ** *p* < 0.01, *** *p* < 0.001 (One-way ANOVA).

Later, we measured the content of metal elements in cocoons after injection and feeding, respectively. We found that both injection and feeding can change the content of metal ions in silk fiber. Figure 3A showed the injection results, which suggests that the contents of Na, Mg, and Cu increased with the dose of EDTA. In addition, K, Fe, and Mn were accumulated in the IEDTA-50 silk fiber. On the other hand, as shown in Figure 3B, the feeding results demonstrated that the content of Na, Zn, Mg, and Mn significantly increased in the FEDTA-500 silk fiber. Ca did not change after feeding with EDTA, but the content of Fe after EDTA feeding was significantly lower than that of the control group. 

These changes can be attributed to the fact that treatment with EDTA disrupts the absorption and utilization of metal ions in the silkworms, and then disorganizes the metal element transport in the silk gland of the silkworms, which finally changes the content of metal elements in silk fiber. Although EDTA mainly chelates divalent metal ions [23], we found that the content of monovalent elements also changed significantly, indicating that there could be ion interactions in the process of metal ions transport to the silk glands. Overall, these data showed that EDTA (injection or feeding) successfully disturbed the content of metal elements in the silk fiber, which indicated that our injection and feeding was effective. Previous in vivo studies have shown that metal ions are involved in the conformational transition of silk protein [15,16,17].

### 2.3. Secondary Structure Analysis

**Figure 4:**
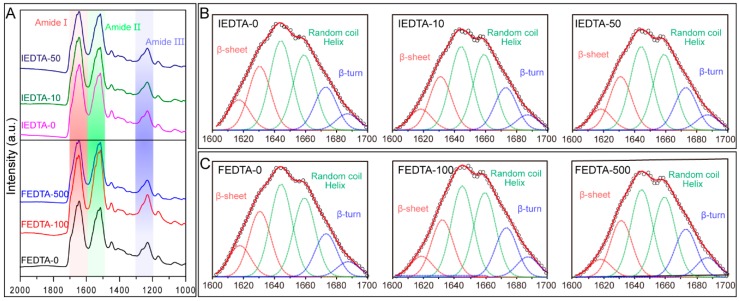
Synchrotron FTIR analysis of silk fiber. (**A**) S-FTIR microspectra of silk fiber with different treatment. Top, injected with EDTA. Bottom, fed with EDTA. (**B**) Deconvolution results of amide I band of injection EDTA silk fiber. (**C**) Deconvolution results of amide I band of feeding EDTA silk fiber. Black circles, original spectra; red solid curve, simulated spectra from the total peaks; dashed curve, deconvoluted peaks.

The overall ionic environment for silk fiber formation was disturbed by EDTA. Previous investigations have shown that many metal ions including Ca^2+^, Mg^2+^, Cu^2+^, Fe^3+^, and Zn^2+^ have an effect on the conformational transitions of silk fibroin [16,17,18,19]. Then, the silk fibers (post injection or feeding) were used for S-FTIR (Synchrotron-Fourier Transform Infrared Spectroscopy) analysis. S-FTIR is usually used to study the secondary structures of silk fiber because of its high brightness, high spatial resolution, high collimation, wide spectral range, and pulse configuration [24,25]. The S-FTIR spectrum of silk fiber has three characteristic regions: amide I (1600–1700 cm^−1^), amide II (1500–1600 cm^−1^), and amide III (1200–1300 cm^−1^) (Figure 4A). The amide I region was selected for secondary structures analysis of silk fiber. By curve fitting and peak fitting, the bands located at 1673–1695 cm^−1^ can be ascribed to β-turn structures and the bands located at 1620 ± 2.0 and 1630 ± 2.0 cm^−1^ can be assigned to the β-sheet structures. The bands located at 1645 and 1660 cm^−1^ can be assigned to the random coil and helix structures, respectively [26] (Figure 4B,C).

Then, we calculated the percentage of each secondary structure. Quantitative analysis of these spectra of the IEDTA silk fiber suggests that IEDTA-0 is composed of 32.29% β-sheet, 53.1% random coil or helix, and 13.56% β-turn structures. IEDTA-10 contains 26.58% β-sheet, 53.03% random coil or helix, and 20.06% β-turn structures. IEDTA-50 contains 28.27% β-sheet, 53.07% random coil or helix, and 18.61% β-turn structures, respectively (Table 1). On the other hand, quantitative analysis of these spectra of the FEDTA silk fiber indicates that FEDTA-0 contains 28.22% β-sheet, 56.8% random coil or helix, and 14.93% β-turn structures. FEDTA-100 is composed of 26.41% β-sheet, 53.13% random coil or helix, and 20.41% β-turn structures. FEDTA-500 contains 26.37% β-sheet, 54.04% random coil or helix, and 19.44% β-turn structures, respectively (Table 1). These data showed that the β-sheet structures in IEDTA and FEDTA silks were apparently lower than those of the control silks. Additionally, we observed that the β-turn structures increased from 13.56% to 20.06% after IEDTA-10 injection. Meanwhile, the β-turn structures increased from 14.93% to 20.41% after FEDTA-100 feeding. These results suggested that metal ions are indeed involved in the conformational transition of silk fibroin in vivo.

Silk fiber is a protein-based high polymer. Its mechanical performance mainly depends on its structure [27]. Silk protein has large molecular weight and contains a variety of amino acids [28]. The main chain carbonyl and amino groups from all the amino acids are able to participate in metal bonding under certain conditions [29]. In practice, only some of the side chain hydroxy groups (Ser, Thr, and Tyr), carboxylate groups (Asp and Glu), and the imidazole group of histidine (His) are able to function as ligands [30]. Fe^3+^ was able to interact with tyrosine residues within the silk. The crosslinking might act as the “bridge” to form the β-sheet structure, thus increasing the mechanical properties of silk fiber [16]. Cu^2+^ and Zn^2+^ can be coordinated by nitrogen atoms present in His imidazole rings to form β-sheet structures [13,14,31]. The Ca^2+^ forms the bonds with oxygen from Asp or Glu residues [32]. In addition, the nature of the metal-donor interaction, that is, the degree of electron sharing, and the bonding strengths are also speculated to play a role in changing the mechanical properties [33].

In this study, we found that the secondary structure composition of silk post EDTA treatment changed significantly, especially the content of β-sheets associated with the strength of silk fiber [26]. We believe that this is due to the alteration in the ionic environment during silk fiber formation, which causes disarray when metal ions interact with amino acids residues, affecting the conformational transition of silk protein, ultimately affecting the mechanical properties of the silk fiber. We consider that this disorder is partial and small as the molecules of silk protein are large and their structure is complex. This quantitative change causes a qualitative change, and this slight change eventually leads to the change in the mechanical properties of the silk fiber.

### 2.4. Mechanical Performance of Silk Fiber

**Figure 5:**
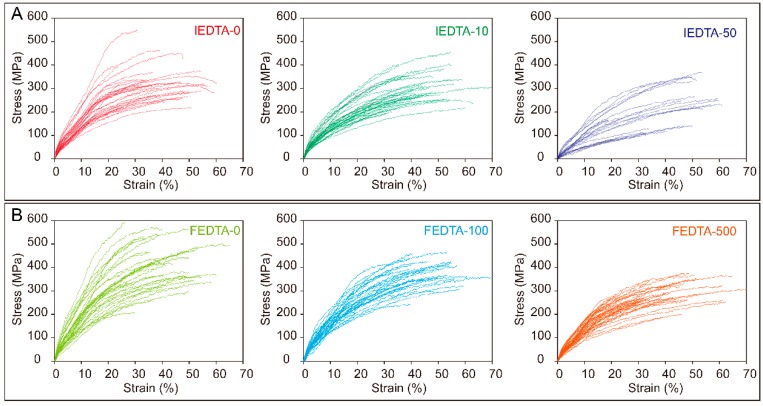
Stress-strain curves of the silk fiber after treatment. (**A**) The silk fiber reeled from silkworms injected of EDTA; (**B**) The silk fiber reeled from silkworms fed with EDTA.

It is known that the mechanical properties of silk fiber are mainly determined by its internal structures [27]. We found that the secondary structures of silk fiber were significantly altered after EDTA injection and feeding. Therefore, the tensile test was carried out to further study the effects of metal ions on the mechanical properties of silk fiber. The stress-strain curves are shown in Figure 5. The figure indicates that the magnitude of mechanical properties of silk fiber, after either injection or feeding, were lower than that of the control groups. The results suggested that changes in the ionic environment could indeed affect the mechanical properties of silk fiber.

**Figure 6:**
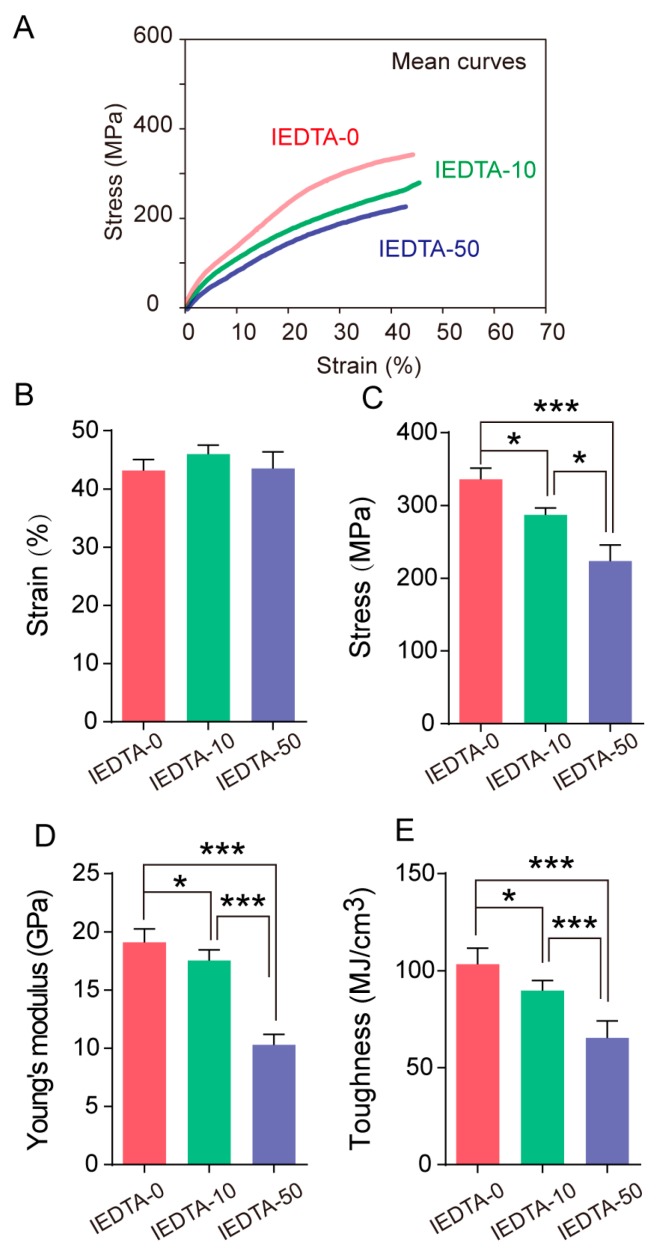
Effect of metal ions on the mechanical properties after injection. (**A**) Average stress-strain curves of each injected group; (**B**) Maximum strain; (**C**) Maximum stress; (**D**) Young’s modulus; (**E**) Toughness. Error bars, SD; * *p* < 0.05, *** *p* < 0.001 (One-way ANOVA).

Then, we further analyzed the mechanical properties of silk fiber. After injecting the silkworms with EDTA, we compared the average stress-strain curves of the silk fiber and found that the magnitude of mechanical properties of silk fiber from IEDTA-10 and IEDTA-50 were lower than that of the control group fiber. Furthermore, comparing the four parameters of mechanical properties (strain, stress, toughness, and Young’s modulus), we found that the stress decreased with the concentration of EDTA (Figure 6). In addition, the Young’s modulus and toughness were lower than that of the control group. Notably, the IEDTA-50 silk fiber had a maximum strength of 223 MPa, a Young’s modulus of 10.29 GPa, and a toughness of 65.35 MJ/cm^3^, which were 33%, 46%, and 37% lower than those of the IEDTA-0 silk fiber. However, the strain of the IEDTA-10 and IEDTA-50 silk fiber remained unchanged.

**Figure 7:**
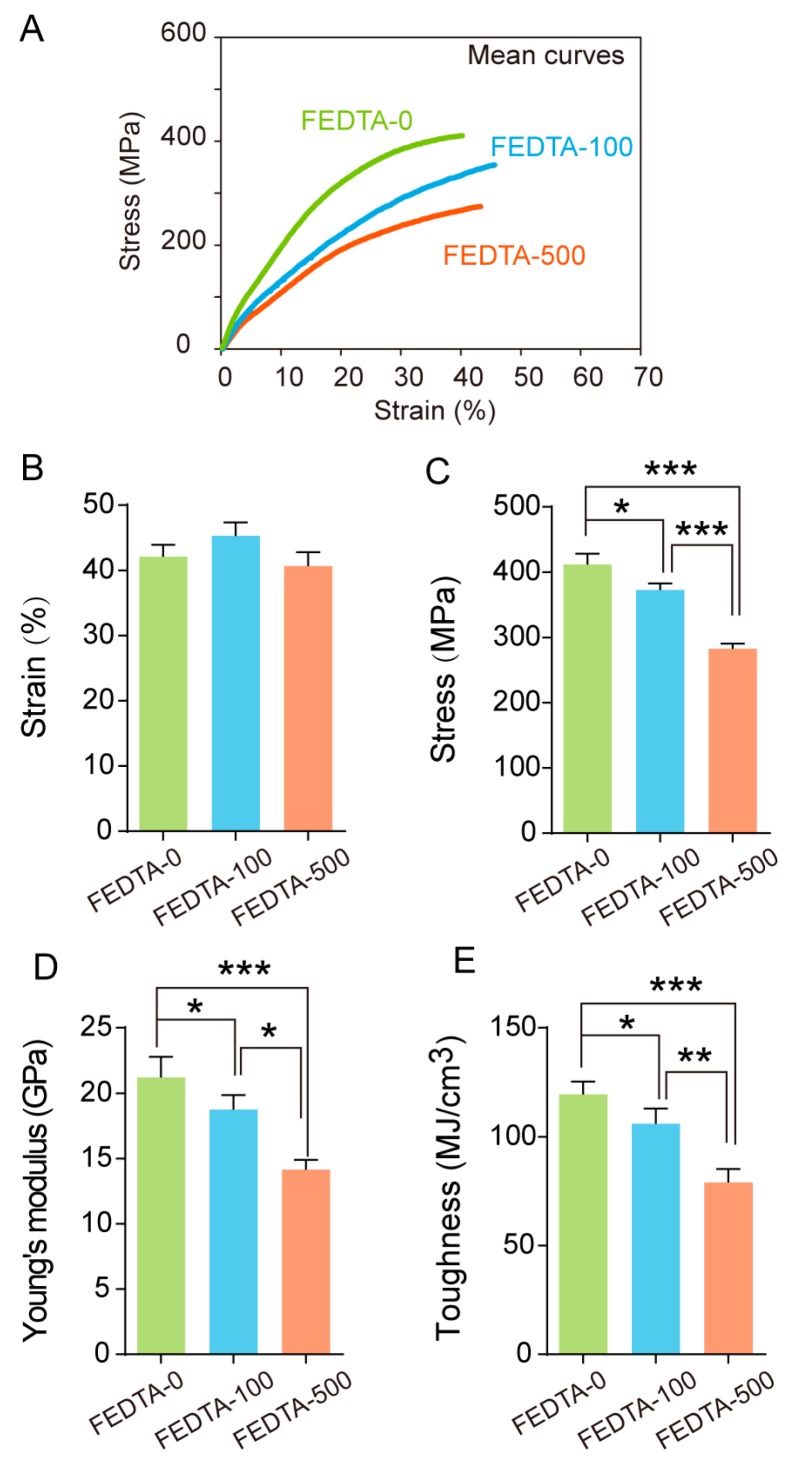
Effect of metal ions on the mechanical properties after feeding. (**A**) Average stress-strain curves of each injected group; (**B**) Maximum strain; (**C**) Maximum stress; (**D**) Young’s modulus; (**E**) Toughness. Error bars, SD; *, *p* < 0.05, **, *p* < 0.01, ***, *p* < 0.001 (One-way ANOVA).

Similarly, the mechanical properties of silk fiber from the FEDTA-100 and the FEDTA-500 group were also lower than that of the control group. In addition, the stress, Young’s modulus, and toughness from the FEDTA-100 and FEDTA-500 groups were, on an average, significantly lower than that of the FEDTA-0 fiber. The FEDTA-500 silk fiber had a maximum strength of 282 MPa, a Young’s modulus of 14 GPa, and a toughness of 78 MJ/cm^3^, which were 31%, 33% and 34% lower than that of the FEDTA-0 silk fiber (Figure 7).

In general, metal ions usually form metal coordination bonds with large biomolecules (especially proteins) [30]. The biological metal coordination interactions have been mostly studied through their roles in essential physiological functions such as catalysis, gas transport, electron transfer, metal scavenging, and signal transduction [34]. However, more recently, metal ions received extensive attention in protein-based biological materials, because the metal coordination complexes endow materials with toughness, hardness, and even autonomic and intrinsic self-healing properties [30,35]. In this paper, all the mechanical properties of the IEDTA and the FEDTA silk fiber were lower than those of the control group silk fiber, except for strain. Previous studies have demonstrated that the change of single metal ions, such as Fe and Cu ions, can increase the mechanical properties of silk fiber [16,17]; these ions are called positive effect ions. In this work, we changed the contents of multiple ions in the silk fiber simultaneously. We found that the contents of Fe and Cu ions in the IEDTA-50 silk fiber increased significantly, but the mechanical properties decreased dramatically. This suggests that when the contents of multiple metal ions with positive effects increase, it does not bring a synergistic effect on the mechanical properties of silk fiber. This phenomenon suggests that the comprehensive mechanical properties of silk fiber require a stability of overall ionic environment. These findings indicate that when studying the effect of metal ions on the mechanical properties of silk fiber, the entire metal ion environment should be taken into consideration. Our findings may help in the development of new silk fiber materials using metal ions in the future.

### 2.5. Current Advances in the Effect of Metal Ions on the Structure and Performance of Silk In Vitro and In Vivo

Natural silk fiber formation depends on the conformational transition of silk fibroin (SF) from random coil or helix to β-sheet [28]. Previous investigations have shown that metal ions can affect the conformational transition of silk fibroin [15,17,36]. Recently, research on the effects of metal ions on the structure and properties of silk has mainly involved in vitro and in vivo. The current research on each metal ion in the silk fiber environment is presented in Table 2. For in vitro, researchers usually add metal ions to regenerated silk fibroin solution to study their effects on the conformation of the silk protein. For in vivo, researchers generally inject metal ion salt solutions or use transgenic methods to disrupt the ionic environment of silk protein formation, and then they analyze the mechanical properties of silk fiber. Previous studies have found that silk glands and silk fiber mainly contain 8 kinds of metal ions, including potassium, sodium, calcium, copper, manganese, magnesium, zinc, and iron [14,15]. Ca^2+^ can induce silk fibroins to form a stable protein network in vitro [15,18,37]. In addition, Ca^2+^ can increase the strength and strain of silk fiber when CaCl_2_ is injected in the transgenic silkworm in vivo [18,19]. However, K^+^ and Na^+^ can break down the silk fibroin network in vitro [15,38]. On the other hand, copper, iron, zinc, and magnesium ions can increase the β-sheet structure of silk fibroin in vitro [14,15,39,40]. Furthermore, injection of FeCl_3_ can increase their strength and toughness [16,36,39]. Injection of CuCl_2_ can increase the strength of silk fiber [17]. However, the effects of Fe^2+^, Mg^2+^, Zn^2+^, and Mn^2+^ on the mechanical properties of silk fiber remain unknown. In general, Ca^2+^, Cu^2+^, and Fe^3+^ can promote the conformational transition of silk fibroin and improve the mechanical properties of silk fiber. These metal ions are called positive effect ions. In contrast, K^+^ and Na^+^ are called negative effect ions.

Previous studies usually focused on individual metal ions changes, while ignoring the effects of the entire metal ion environment. In this paper, we changed the overall ionic environment, and found that although the content of certain positive effect metal ions increased (Figure 3), the final mechanical properties of the silk fiber decreased. The strength and toughness of IEDTA-50 silk decreased by 33% and 37%, respectively (Figure 6C,E). The strength, Young’s modulus, and toughness of IEDTA-50 silk decreased by 31%, 33%, and 34%, respectively (Figure 7). These results might be due to the interactions of entire metal ions, such as the regulation of ion transporters or the competition of amino acid residue binding sites. Many ion-transporting proteins, such as Na^+^, K^+^, Ca^2+^, Zn^2+^, Cu^2+^, and Fe^3+^ transporters, were highly expressed in the silk glands [17,42]. Overexpressing the calcium-transporting protein sarcoplasmic/endoplasmic reticulum Ca^2+^ ATPase (SERCA) in the spinning duct of the silkworm decreased the Ca^2+^ content in silk significantly, and these fiber became stronger, stiffer, and tougher [19]. Moreover, many ion transporters can transport multiple ions, such as transferrin can transport Fe, Cu, and Zn ions [43]. On the other hand, many studies have indicated that silk fibroin could have many potential metal ion binding sites, such as tyrosine (Tyr), histidine (His), and asparagine (Asn) residues in the heavy chain of the hydrophilic spacer GTGSSGFGPY–VAN(H)GGYSGYEYAWSSESDFGT [36,44]. K^+^ can stabilize the state of tyrosine phenolic-OH [38]. And K^+^ also might interact with the negatively charged residues, such as aspartate and glutamate [17]. Cu^2+^ and Zn^2+^ have the ability to form complexes with histidine residues to induce β-sheet formation and aggregation [13,14]. Ca^2+^ is able to form a complex with irregular geometry ligands [45], and Mg^2+^ mainly forms octahedral complexes [46]. Moreover, our previous studies revealed that Fe^3+^ was able to interact with tyrosine and tryptophan residues, acting as the “bridge” to form the β-sheet structure [16]. These findings indicate that metal ions competitively interact with amino acid residues. Additionally, polarizability (the ratio of charge to ionic radius) is a main factor for the strength of metal ion complex. Alkaline metal ions like Ca^2+^ and Mg^2+^ interact with ligands stronger than alkali metal ions because of increased nuclear charge, which also applies to divalent cations, such as Mn^2+^ > Fe^2+^ > Cu^2+^ > Zn^2+^ [30]. In proteins, the metal ion’s interaction with ligands not only depends on the potential ligands, but also their stereochemical arrangement, and the relative sizes of the metal ions [47]. Our study disturbed the entire ions environment in hemolymph and silk gland for silk fiber formation. We believe that changes in the entire metal ion environment can affect how the metal ions interact with amino acid residues, which can result in the secondary structure and mechanical properties of silk fiber being changed significantly. Overall, these results indicated that the effect of metal ions on the mechanical properties of silk is synergistic in the case of multiple metal ions. Subsequent research on the effect of metal ions on the structure and properties of silk in the future should take the entire metal ion interactions into consideration.

## 3. Materials and Methods

### 3.1. Silkworms

Silkworms (strain 21-872) were obtained from the Biological Science Research Center, Southwest University, China. The larvae were raised in lab conditions (27 ± 1 °C, 75% relative humidity (RH)) and fed with mulberry leaves until the fifth instar. The fifth instar larvae were used for feeding and injection with EDTA·2Na·2H_2_O. EDTA (Sangon Biotech, Shanghai, China) is sparingly soluble in water. Hence, to avoid the influence of solvent on injection and feeding, we chose EDTA·2Na·2H_2_O dissolved in water for our experiments. For the convenience of description, it will be referred to as “EDTA” in the subsequent sections.

### 3.2. Experimental Design

#### 3.2.1. Injecting EDTA to Silkworms (IEDTA)

The fifth instar larvae on day 1 were divided into three groups (each group had 60 larvae) for injection. Ten microliters of different doses (10 and 50 mM) of EDTA solutions were injected into the hemolymph of the larvae (IEDTA-10 and IEDTA-50). In order to reduce the damage to the larvae, injection was performed on days 1, 3, 5, and 7 of the fifth instar. The spiracles in the postmedian of the silkworm were used for injection. Control group (*n* = 60) was injected with 10 μL ddH_2_O (IEDTA-0).

#### 3.2.2. Feeding EDTA to Silkworms (FEDTA)

A total of 180 larvae in the fifth instar (on day 1) were divided into three groups. The larvae were fed with mulberry leaves treated with two concentrations of EDTA (100 and 500 mM) every day until day 7 (FEDTA-100 and FEDTA-500). The EDTA solutions were uniformly sprayed on the fresh mulberry leaves using a spray bottle. The control group (*n* = 60) was fed with normal mulberry leaves (FEDTA-0) until spinning. All the fifth instar larvae were reared under the conditions of 24–25 °C and 70–75% RH until spinning. The cocoons were collected on the fifth day after silkworm spinning.

### 3.3. Elemental Analysis

To confirm the effect of EDTA, we chose the hemolymph and silk gland of silkworms in the wandering stage to perform the elemental analysis. The cocoons were randomly collected and dried thoroughly in an oven at 65 °C for elemental analysis. The dried cocoons were firstly cut into small pieces, dissolved in 15 mL HNO_3_ (Kelong, Chengdu, China) solution, and then incubated in an oven at 140 °C for 4 h. Later, they were diluted with deionized water to 25 mL. When the silkworm just started spinning, we dissected the silkworm and extracted the hemolymph and silk gland, simultaneously. The hemolymph and the silk gland samples were also processed as above. The content of metal elements in the hemolymph and silk gland were determined by using the same group of silkworms. The silk fiber metal element content was determined by using the rest of the same group of silkworm cocoons. All tests were performed on the same treatment group. The Z-5000 Inductive coupling plasma atomic absorption spectroscopy (ICP-AAS) (Hitachi, Tokyo, Japan) was used for measuring the metal elemental content according to the N_2_O-acetyleneflame AAS method. All the tests were repeated three times.

### 3.4. FTIR Spectroscopy of a Single Silk Fiber

Degummed silk fiber was used for FTIR analysis. The collected cocoons were first boiled twice in 0.5% (*w*/*v*) NaHCO_3_ (Sangon Biotech) solution for 30 min for degumming, and then were rinsed with deionized water. The degummed silk fiber was dried thoroughly at room temperature. The experiments were performed at BL01B in the Shanghai Synchrotron Radiation Facility (SSRF). Infrared spectra were recorded using the Nicolet 6700 Fourier transform infrared spectrometer with infrared microscopy and imaging systems. For every measurement, the infrared spectra were collected in the mid-infrared (MIR) range of 800–3800 cm^−1^ at a resolution of 4 cm^−1^ with 256 coadded scans.

### 3.5. Mechanical Testing of Silk Fiber

The single silk fiber was reeled from the cocoons in hot 0.5% (*w*/*v*) NaHCO_3_ solution. First, 50 mm of the single fiber was collected and cut into two fragments of lengths 20 and 30 mm, respectively. The 20 mm segment was used for diameter measurement, the 30 mm segment for the mechanical testing, respectively. The 20 mm segments were sputter coated in MP-19020NCTR Neocoater (JEOL, Tokyo, Japan) and then transferred to the JCM-5000 scanning electron microscope (JEOL) for diameter measurement. The single fiber with two brins was approximated as two small circles. Then, the diameters were used to calculate the cross-sectional areas of the small circles as the cross-sectional areas of single fiber. The diameter was used to calculate the cross-sectional areas of the single fiber. The initial length of the single fiber was measured using a caliper before mechanical testing. Tensile test was carried out using the single fiber on an AG-X plus instrument (Shimadzu, Kyoto, Japan) with a stretch speed of 2 mm/min. Each group measured 40 fibers for statistical analysis. The strain-stress curves were measured according to the method previously reported [18].

### 3.6. Statistical Analysis

All experiments were replicated three times, the data are presented as the mean and standard deviation (SD), and they were analyzed using GraphPad Prism software (GraphPad, La Jolla, CA, USA). One-way analysis of variance (ANOVA) was performed between means to determine the significant differences using Tukey’s multiple comparisons test (*p* < 0.05).

## 4. Conclusions

In this paper, we investigated the effects of metal ions on the mechanical properties of silk fiber through directly disturbing the ionic environment for silk fiber formation by EDTA. ICP-AAS results indicated that EDTA changed the overall metal ion environment successfully. Synchrotron FTIR showed that the secondary structure of the silk fiber changed significantly, especially the content of β-sheet and β-turns. Mechanical testing showed that the magnitude of various mechanical properties of silk fiber significantly decreased after injection or feeding with EDTA. Specifically, the mechanical properties of the IEDTA-50 and FEDTA-500 silk fiber significantly decreased in strength, Young’s modulus, and toughness. This research showed that the overall ionic environment is crucial for the formation of silk fiber with favorable mechanical properties. This investigation provided a new understanding of the relationship between the overall metal ion environment and the mechanical properties of silk fiber. These findings might also prove to be helpful in expanding the applications of silk fiber in various fields by helping us modify their mechanical properties by altering metal ion environment.

## Figures and Tables

**Table 1:** Averaged percentages of secondary structures of silk fiber after EDTA injection and feeding.

Secondary Structures (%)	Injecting EDTA			Feeding EDTA		
	0 mM	10 mM	50 mM	0 mM	100 mM	500 mM
β-sheet	32.29 ± 1.41	26.85 ± 1.60	28.27 ± 1.7	28.22 ± 3.0	26.4 ± 1.5	26.37 ± 2.8
Random coil and Helix	53.1 ± 2.58	53.25 ± 1.0	53.25 ± 1.2	56.8 ± 5.0	53 ± 0.6	54 ± 1.1
β-turn	13.56 ± 0.80	20.06 ± 2.2	18.6 ± 1.0	14.93 ± 3.0	20.4 ± 1.0	19.4 ± 2.0

**Table 2:** Current research on the effect of metal ions on silk structure and performance in vitro and in vivo.

Metal Ions	In Vitro	In Vivo
	Secondary Structure	Mechanical Performance
Ca^2+^	α-helical intermediate conformation [37]	Injection of CaCl_2_: strain increased 30.9% [18]
	Forming a stable protein network [15,18]	Transgenic silkworm: strength/strain increased 21.1%/73.1% [19]
Na^+^	Break down the protein network [15]	Transgenic silkworm: the average tenacity, Young’s modulus, and toughness of the transgenic silks decreased significantly [19]
K^+^	Decrease β-sheet like conformation and break down the protein network [15,38]	Injection of KCl: strength increased 71.4% [17]
Cu^2+^	β-sheet increased [13,15,17]	Injection of CuCl_2_: strength increased 51.9% [17]
Fe^2+^	Less influence [36]	Not reported
Fe^3+^	β-sheet increased [16,36,39]	Injection of FeCl_3_: strength/toughness increased 13%/29% [16]
Mg^2+^	β-sheet increased [15]	Not reported
Zn^2+^	β-sheet increased [15,40]	Not reported
Mn^2+^	Stabilizing the Silk I state [41]	Not reported

## Abbreviations

EDTA	Ethylenediaminetetraacetic acid
EDTA·2Na·2H_2_O	Ethylenediaminetetraacetic acid disodium salt
FTIR	Fourier Transform Infrared Spectroscopy
